# Supplementary material for: Prospective pilot study on the predictive significance of plasma miR-30b-5p through the study of echocardiographic modifications in Cavalier King Charles Spaniels affected by different stages of myxomatous mitral valve disease: The PRIME study
Source: PLoS One. 2022 Dec 27;17(12):e0274724. doi: 10.1371/journal.pone.0274724 (PMC9794076; doi:10.1371/journal.pone.0274724)
Supplement: S1 Table — E = E wave velocity, EDVI = end-diastolic volume index, ESVI = end-systolic volume index, FS = fractional shortening, LA/Ao = left atrial-to-aortic root ratio, LVIDdN = left ventricular end-diastolic diameter normalized for body weight, Murmur = systolic heart murmur intensity. 95% Lower limit and 95% Upper limit: Lower and upper limits of 95% confidence interval for the mean marginal effect. (DOCX) [file pone.0274724.s001.docx]

**Table S1. Estimates of marginal effects for continuous and ordinal variables.**

| **Continuous responses** | | | | | |
| --- | --- | --- | --- | --- | --- |
|  | Estimate | Standard error | P | 95% Lower limit | 95% Upper limit |
| **LVIDdN (cm/kg)** | -0.0004 | 0.0001 | 0.0003 | -0.0006 | -0.0002 |
| **FS (%)** | -0.0052 | 0.0037 | 0.1685 | -0.0126 | 0.0021 |
| **E (m/s)** | -0.0002 | 0.0001 | 0.0560 | -0.0004 | 0.0000 |
| **LA/Ao** | -0.0004 | 0.0002 | 0.0484 | -0.0007 | 0.0000 |
| **EDVI (ml/m^2^)** | -0.0503 | 0.0144 | 0.0005 | -0.0785 | -0.0220 |
| **ESVI (ml/m^2^)** | -0.0124 | 0.0045 | 0.0055 | -0.0211 | -0.0036 |
| **Ordinal responses** | | | | | |
|  | Estimate | Standard error | P | 95% Lower limit | 95% Upper limit |
| **Murmur** | 0.001001 | 0.000676 | 0.069334 | -0.000324 | 0.002326 |
| **Regurgitant jet size** | 0.000059 | 0.000131 | 0.326218 | -0.000198 | 0.000316 |
| **MINE score** | 0.000949 | 0.000934 | 0.154800 | -0.000882 | 0.002780 |

Estimates of marginal effects of miR-30b-5p on the response variables. E = E wave velocity, EDVI = end-diastolic volume index, ESVI = end-systolic volume index, FS = fractional shortening, LA/Ao = left atrial-to-aortic root ratio, LVIDdN = left ventricular end-diastolic diameter normalized for body weight, Murmur = systolic heart murmur intensity. 95% Lower limit and 95% Upper limit: lower and upper limits of 95% confidence interval for the mean marginal effect.
